# The Hippo Signaling Core Components YAP and TAZ as New Prognostic Factors in Lung Cancer

**DOI:** 10.3389/fsurg.2022.813123

**Published:** 2022-03-21

**Authors:** Yu Jiang, Wen-Jing Xie, Rong-Wei Chen, Wei-Wei You, Wei-Lin Ye, Hong Chen, Wen-Xu Chen, Jian-Ping Xu

**Affiliations:** ^1^Department of Clinical Laboratory, Fuzhou Second Hospital Affiliated to Xiamen University, Fuzhou, China; ^2^Department of Clinical Laboratory Medicine, Fujian Medical University, Fuzhou, China

**Keywords:** non-small cell lung cancer, Hippo pathway, YAP, TAZ, prognosis

## Abstract

**Background:**

The Hippo pathway is an essential signaling cascade that regulates cell and organ growth. However, there is no consensus about (i) the expression levels of the Hippo signaling core components yes-associated protein (YAP) and transcriptional co-activator with PDZ-binding motif (TAZ) in lung cancer, especially in small cell lung cancer (SCLC), or (ii) their association with the prognosis of patients with SCLC.

**Methods:**

We screened relevant articles and identified eligible studies in the PubMed, EMBASE, COCHRANE, and WanFang databases. A combined analysis was performed to investigate (i) the expression levels of the major effectors, YAP and TAZ, in lung cancer and its subsets and (ii) their prognostic role in lung cancer, especially in SCLC.

**Results:**

In total, 6 studies related to TAZ and 13 studies concerning YAP were enrolled in this meta-analysis. We found that high TAZ expression was significantly associated with poor overall survival (OS) of patients with non-small cell lung cancer (NSCLC) in the overall population [*P*_h_ < 0.001, crude hazard ratio (HR) = 1.629, 95% CI = 1.199–2.214 for *TAZ* expression; *P*_h_ = 0.029, adjusted HR = 2.127, 95% CI = 1.307–3.460 for TAZ], the Caucasian population (*P*_h_ = 0.043, crude HR = 1.233, 95% CI = 1.030–1.477 for *TAZ* expression), and the Asian population (*P*_h_ = 0.551, adjusted HR = 2.676, 95% CI = 1.798–3.982 for TAZ). Moreover, there was a significant negative association between YAP expression and an unsatisfactory survival of patients with lung cancer (*P*_h_ = 0.327, crude HR = 1.652, 95% CI = 1.211–2.253 for *YAP* expression) and patients with NSCLC [disease-free survival (DFS): Ph = 0.693, crude HR = 2.562, 95% CI = 1.876–3.499 for *YAP* expression; Ph = 0.920, crude HR = 2.617, 95% CI = 1.690–4.052 for *YAP*-mRNA; OS: Ph = 0.878, crude HR = 1.777, 95% CI = 1.233–2.562 for YAP expression], especially in the Asian population (DFS: *P*_h_ = 0.414, crude HR = 2.515, 95% CI = 1.755–3.063; OS: *P*_h_ = 0.712, crude HR = 1.772, 95% CI = 1.214–2.587). However, no association was observed in the multivariate combined analysis. High YAP expression was significantly associated with short OS of patients with SCLC in our combined multivariate analysis in the Asian population (*P*_h_ = 0.289, crude HR = 4.482, 95% CI = 2.182–9.209), but not with crude data (*P*_h_ = 0.033, crude HR = 1.654, 95% CI = 0.434–6.300).

**Conclusion:**

The Hippo pathway is involved in carcinogenesis and progression of NSCLC and SCLC, and high expression levels of YAP and TAZ are independent and novel prognostic factors for lung cancer.

## Introduction

Lung cancer is a primary malignancy with one of the highest rates of morbidity and mortality in China (1). According to its histological classification, lung cancer is stratified into small cell lung cancer (SCLC) and non-small cell lung cancer (NSCLC), of which the latter subgroup accounts for ~80% of lung cancer cases (2). While emerging molecular-targeted and onco-immunological therapeutics are increasingly used in the clinic (2, 3), the survival rates of patients need to be further improved.

The Hippo pathway is an important signaling cascade related to cell proliferation, apoptosis, and differentiation. Yes-associated protein 1 (YAP1) and transcriptional co-activator with PDZ-binding motif (TAZ) are transcription co-activators and the core downstream effectors of the Hippo signaling pathway (4). When the Hippo pathway is activated, the two molecules are phosphorylated and retained in the cytoplasm in normal cells. On the contrary, YAP/TAZ can translocate into the nucleus to activate the transcription of multiple oncogenes in tumor cells (5). Meanwhile, they are frequently upregulated and biologically function as oncogenes *via* participation in multiple cancer-related processes, including cancer cell growth, migration, and distal metastasis in various malignancies, such as colorectal, gastric, and lung cancer (6–8).

Several studies have shown that the Hippo pathway is activated upon carcinogenesis and lung cancer progression (9–11). However, different YAP and TAZ expression levels have been reported in lung cancer and its subsets. Moreover, no consensus has been reached with respect to their prognostic values in patients with lung cancer. Yang et al. reported that YAP was not associated with the disease stage and survival of patients with SCLC (12). In another study, higher expression of YAP1 was associated with worse progression-free survival in patients with epidermal growth factor receptor (*EGFR*)-mutant NSCLC treated with first-line EGFR tyrosine kinase inhibitors (13). Although higher TAZ mRNA and protein levels were associated with shorter survival of patients with NSCLC (14), the nuclear localization of TAZ was increased, which was correlated with poor prognosis in lung squamous cell carcinomas, but not lung adenocarcinomas (15).

To better understand the prognostic role of the Hippo pathway in lung cancer, we investigated YAP and TAZ expression in lung cancer and analyzed their associations with the prognosis of patients with lung cancer in this meta-analysis.

## Materials and Methods

The Medical Ethics Committee of Fuzhou Second Hospital affiliated with Xiamen University approved this study. To obtain studies for our meta-analysis, we screened and identified the relevant articles according to the Preferred Reporting Items for Systematic Reviews and Meta-Analyses (PRISMA) guidelines (16). First, the following words and phrases were used to identify candidate articles in the PubMed, EMBASE, COCHRANE, and WanFang databases: “lung cancer,” “lung squamous cell carcinomas,” “lung adenocarcinomas,” “non-small cell lung cancer,” “small cell lung cancer,” “YAP1,” “YAP,” “Yes-associated protein,” “Hippo pathway,” “transcriptional co-activator with PDZ-binding motif,” “TAZ,” “WWTR1,” “survival,” “outcome,” “prognosis,” “progression-free survival,” “recurrence-free survival,” “disease-free survival,” and “overall survival.” The retrieval deadline was August 30, 2021. We also screened references of the relevant reports to obtain additional studies. After reading the title and abstract of the candidate articles, we identified the relevant studies. Finally, we identified eligible studies by reading the full texts of the relevant articles. The inclusion criteria were as follows: (1) the study reported the relationship between YAP or TAZ and prognosis of lung cancer and (2) the study provided clinical baseline characteristics and reported the results in hazard ratios (HRs) and 95% CIs. Articles that did not provide detailed survival data and comments were excluded, along with letters, reviews, and meta-analyses.

The following clinical baseline characteristics were extracted from each study: the first author's name, study design, country, race, recruitment time, disease, number of included cases, treatment, outcome, HR, and 95% CI. All data were rechecked.

The HR and 95% CI were selected as parameters to assess the strength of the association between YAP1 or TAZ and the prognosis of the cases with lung cancer. The Q test and estimated I^2^ were selected to evaluate the heterogeneity of included studies in this meta-analysis, where *P*_h_ < 0.1 or I^2^ > 50% was considered to indicate substantial heterogeneity. The combined analysis of eligible studies was assessed by the Z test based on a fixed (*P*_h_ > 0.1) or random (*P*_h_ < 0.1) model. Publication bias within the included studies was evaluated by funnel plots (17). The Stata v.11.0 software (Stata Corporation, College Station, TX, USA) was used for all statistical analyses, and *p* < 0.05 was considered to indicate statistical significance.

## Results

**Figure 1:**
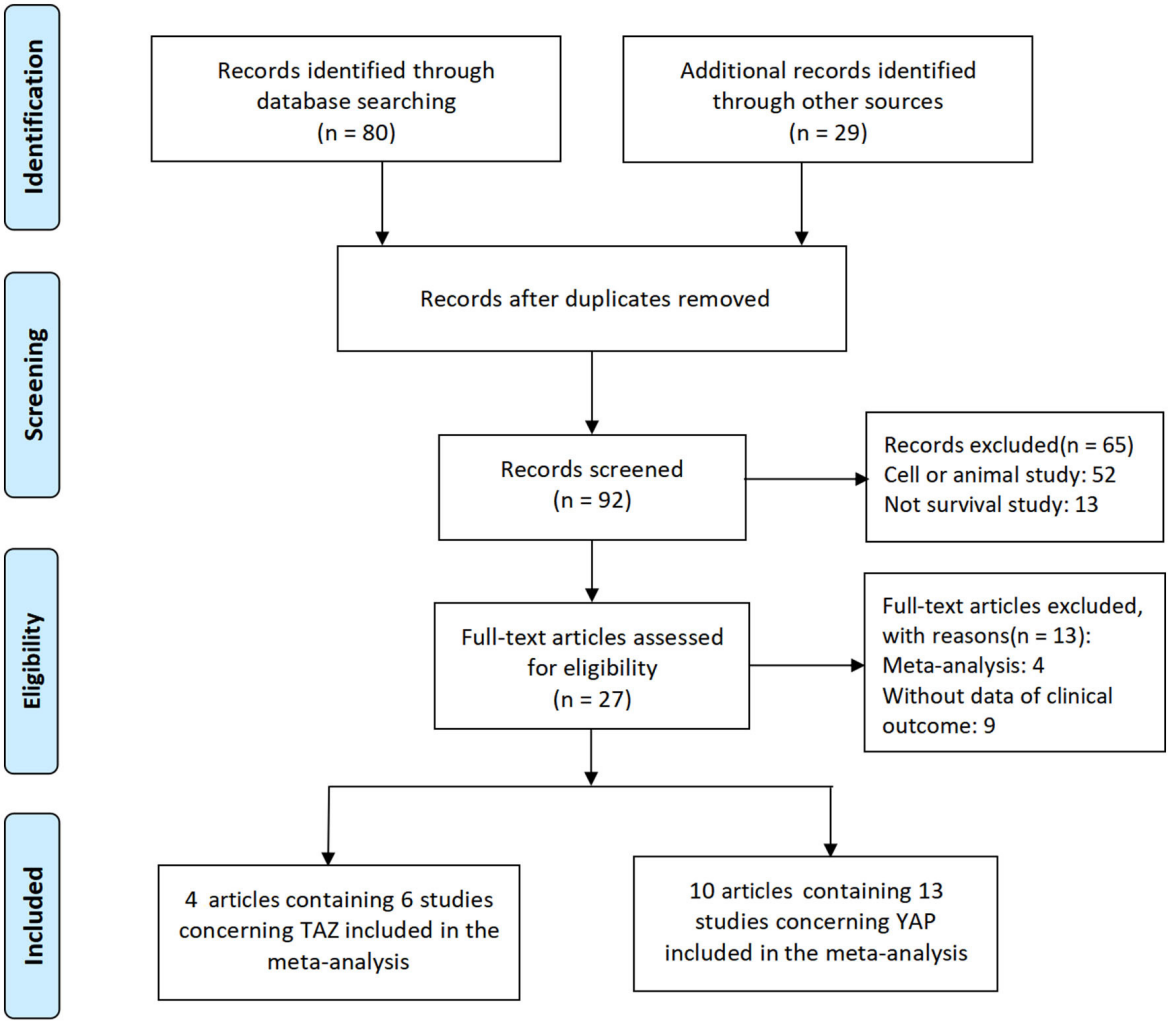
The screening and identification of studies included in this meta-analysis.

The detailed search and selection procedure of eligible studies is described in Figure 1. Initially, a total of 109 articles were selected. After the exclusion of duplicated articles, unrelated research, and other studies that did not meet the inclusion criteria, 14 articles, including 19 studies, were included in this meta-analysis (13–15, 18–28).

The detailed baseline characteristics of these studies are described in Table 1. Among the 14 included articles, 4 articles including 6 studies (14, 15, 23, 28), and 10 articles (13, 18–22, 24–27) containing 13 studies reported the relationship between TAZ and YAP and the prognosis of patients with lung cancer, respectively. Three studies, including 350 cases, reported these associations concerning SCLC in the Chinese population (24, 26), and the other 16 studies, including 857 cases, reported these relationships concerning NSCLC. Three studies were conducted in the Caucasian population (13, 14, 21), and the others were performed in the East Asian population. Most of the studies reported YAP and TAZ protein expression in lung cancer. However, three studies reported mRNA levels (13, 14, 21).

**Table 1:** The baseline characteristics of eligible study.

**References**	**Year**	**Region**	**Race**	**Time**	**Number**	**Cancer**	**Stage**	**Therapy**	**Biomarker**	**Detection**	**Cut-off**	**Outcome**
Wang et al. (27)	2010	China	Asian	1995–2013	98	NSCLC	I-IV	NO	Cytoplasm, nucleus YAP	IHC	Not available	OS
Xie et al. (28)	2012	China	Asian	2003–2005	181	NSCLC	I-III	Surgery	TAZ	IHC	TAZ-negative: staining *H* score 0–50, TAZ-positive: staining *H* score >50	OS
Noguchi et al. (14)	2014	Sweden	Caucasian	1995–2005	1,707	NSCLC	I-III	Surgery	TAZ Its mRNA	IHC; mRNA chip	TAZ-negative: staining score 0–6, TAZ-positive: staining score >6	OS
Sun et al. (25)	2014	Korea	Asian	2003–2008	241	LUAD	I-III	Surgery EGFR-TKI	Cytoplasm, nucleus YAP	IHC	YAP-negative: cytoplasmic reactivity <50%; YAP-positive: cytoplasmic reactivity ≥ 50	OS
Kim et al. (22)	2015	Korea	Asian	2008–2012	167	LUAD	I-IV	Surgery	Cytoplasm, nucleus YAP	IHC	YAP-negative: cytoplasmic reactivity <50%, nuclear staining <10%; YAP-positive: cytoplasmic reactivity ≥ 50, nuclear staining ≥ 10%;	DFS OS
Malik et al. (23)	2017	India	Asian	2013–2015	69	NSCLC	I-III	Surgery	TAZ	Western blotting	NA	OS
Chaib et al. (13)	2017	European China	Caucasian Asian	Not available	119	NSCLC	III-IV	EGFR-TKI	YAP1 mRNA	qPCR	YAP-negative: cytoplasmic reactivity <50%; YAP-positive: cytoplasmic reactivity ≥ 50	DFS
Hong et al. (20)	2018	Korea	Asian	2010–2017	63	LUAD	III-IV	EGFR-TKI	Cytoplasm, nucleus YAP	IHC	TAZ-negative: staining *H* score 0–100, TAZ-positive: staining *H* score > 100	DFS OS
Karachaliou et al. (21)	2018	Spain	Caucasian	Not available	17	NSCLC	IV	Nivolumab	YAP	YAP mRNA	YAP-negative: ≤ median, YAP-positive: >median	DFS OS
Wang et al. (15)	2019	China	Asian	2012–2016	139	LUSC LUAD	I-IV	Not available	Nuclear TAZ	IHC	TAZ-negative: positive tumor cell ≤ 10%, TAZ-positive: positive tumor cells > 10%.	OS
Song et al. (24)	2019	China	Asian	2008–2012	53	SCLC		Not available	Cytoplasm, nucleus YAP	IHC	TAZ-negative: staining score 0–2, TAZ-positive: staining score > 2	OS
Chen et al. (18)	2019	China	Asian	1998–2004	102	NSCLC	I-III	Surgery	Cytoplasm, nucleus YAP	IHC	YAP-negative: staining score 0–150, YAP-positive: staining score > 150	DFS OS
Deng et al. (19)	2020	China	Asian	2015–2016	50	NSCLC	I-III	Surgery	YAP	IHC	YAP-negative: staining score 0–6, YAP-positive: staining score > 6	DFS
Wang et al. (26)	2021	China	Asian	2005–2006	297	pSCLC cSCLC	I-IV	Surgery	YAP	IHC	YAP-negative: H-score <10, YAP-positive: H-score ≥ 10	DFS OS

*LUSC, lung squamous cell carcinomas; LUAD, lung adenocarcinomas; NSCLC, non-small cell lung cancer; SCLC, small cell lung cancer; pSCLC, pure small cell lung cancer; cSCLC, combined small cell lung cancer; IHC, Immunohistochemistry; qPCR, quantitative-polymerase chain reaction; DFS, disease-free survival; OS, overall survival*.

**Figure 2:**
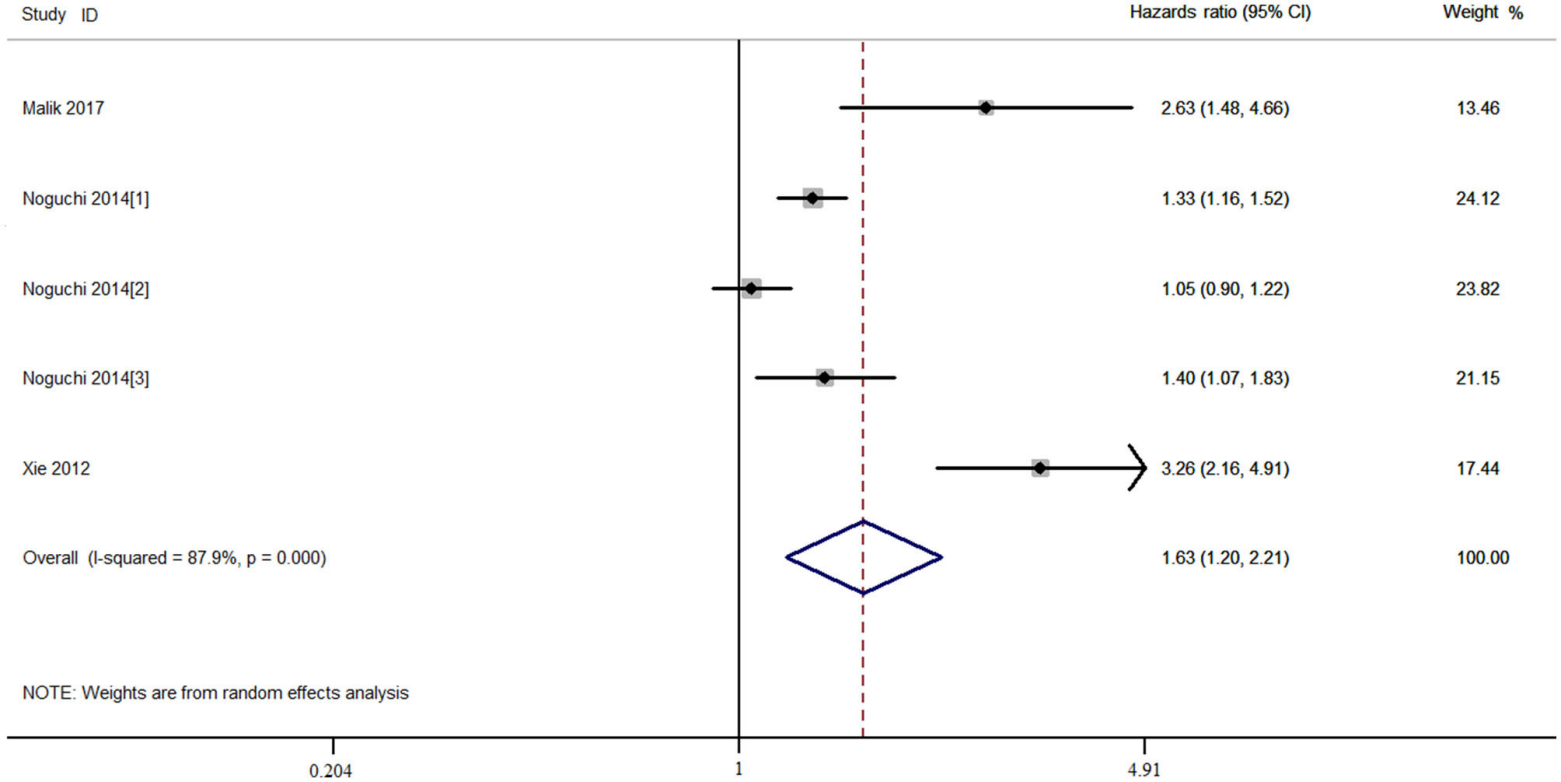
Combined meta-analysis between transcriptional co-activator with PDZ-binding motif (TAZ) expression and overall survival of patients with non-small cell lung cancer (NSCLC).

The combined HR and 95% CI of TAZ expression and overall survival (OS) of patients with NSCLC are described in Figure 2 and Table 2. TAZ expression and OS of patients with NSCLC were reported for a total of 1,977 patients over five studies. Two studies reported *TAZ* mRNA levels, and three studies were conducted in the Asian population. The crude and adjusted HR and 95% CI were extracted from five and three studies, respectively. The combined results showed that high TAZ expression was significantly associated with poor OS in the overall population (*P*_h_ < 0.001, I^2^ = 87.8%, crude HR = 1.629, 95% CI = 1.199–2.214 for TAZ and *TAZ*; *P*_h_ = 0.029, I^2^ = 66.7%, adjusted HR = 2.217, 95% CI = 1.307–3.460 for TAZ), the Caucasian population (*P*_h_ = 0.043, I^2^ = 68.3%, crude HR = 1.233, 95% CI = 1.030–1.477), and the Asian population (*P*_h_ = 0.551, I^2^ = 0.00%, adjusted HR = 2.676, 95% CI = 1.798–3.982).

**Table 2:** The combined prognostic results of TAZ in the meta-analysis.

**Biomarker**	**Disease**	**Survival**	**Crude HR and 95%CI**						**Adjusted HR and 95%CI**					
			**Race**	**Sample** **size**	**Eligible/** **study**	***P_h_*****-value** **I^2^**	**Random** **model**	**Fixed** **model**	**Race**	**Sample** **size**	**Eligible** **study**	***P_h_*****-value** **I^2^**	**Random** **model**	**Fixed** **model**
TAZ expression	NSCLC	OS	Overall	1,977	5	<0.001 87.8%	1.629 (1.199–2.214)	1.307 (1.194–1.432)	–	–	–	–	–	–
TAZ expression	NSCLC	OS	Caucasian	1,727	3	0.043 68.3%	1.233 (1.030–1.477)	1.221 (1.111–1.343)	–	–	–	–	–	–
TAZ	NSCLC	OS	Overall	595	3	0.002 84.4%	2.238 (1.240–4.039)	1.903 (1.543–2.347)	Mixed	734	4	0.029 66.7%	2.127 (1.307–3.460)	1.681 (1.339–2.111)
TAZ	NSCLC	OS	Asian	389	3	–	–	–	Asian	389	3	0.551 0.0%	2.676 (1.798–3.982)	2.676 (1.798–3.982)

*TAZ means TAZ protein expression detecting by immunohistochemistry; TAZ expression includs TAZ protein expression detecting by immunohistochemistry and TAZ-mRNA transcription; NSCLC, non-small cell lung cancer; overall population includes Asians and Caucasian cases; HR, hazards ratio; CI, confidence interval; P_h_-value, P-value of heterogeneity; OS, overall survival*.

**Figure 3:**
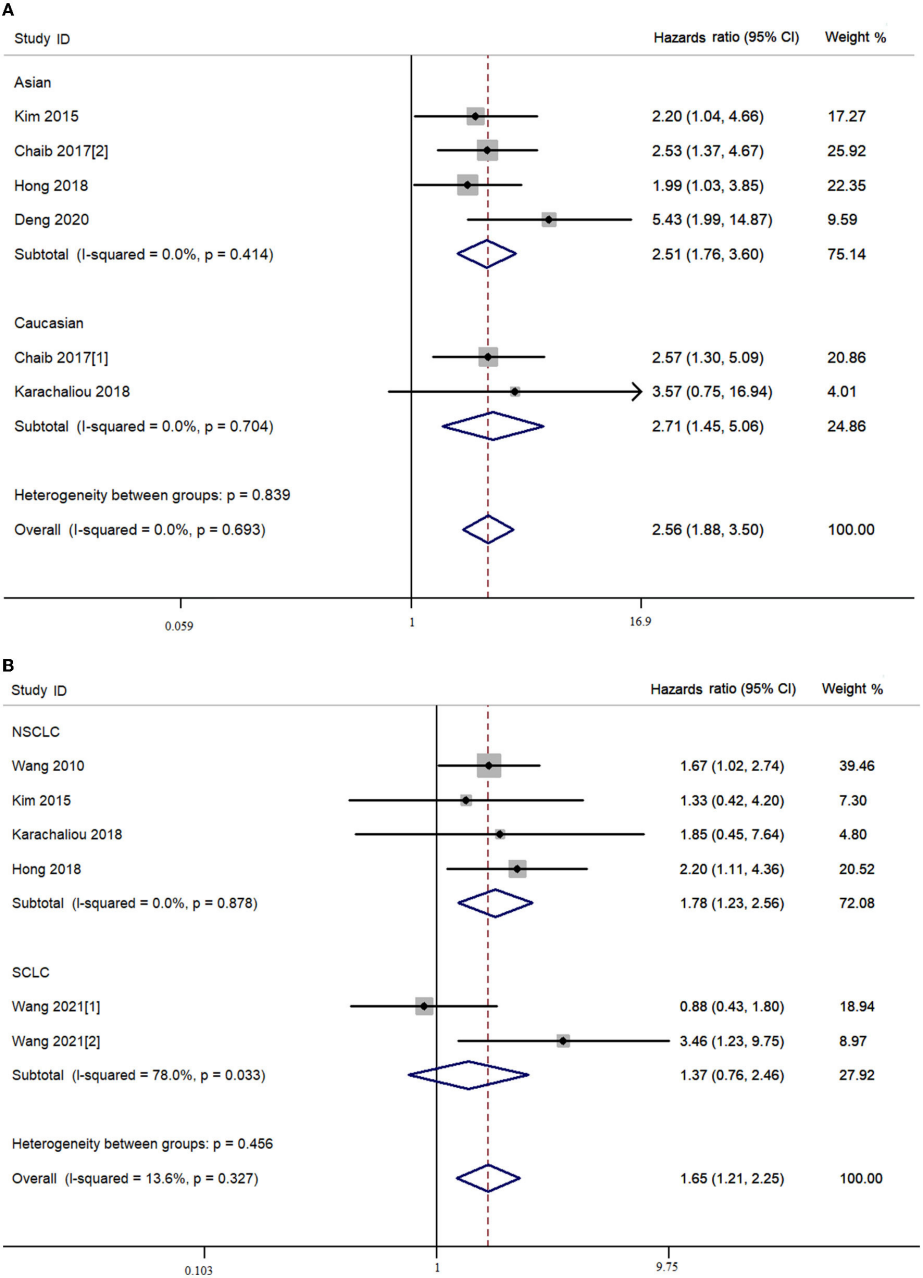
Combined meta-analysis between Yes-associated protein (YAP) expression and survival of lung cancer. **(A)** Disease-free survival. **(B)** Overall survival.

In the combined analysis of YAP expression and survival of patients with lung cancer, the OS of the patients with lung cancer with high YAP expression was significantly shorter than in patients with low YAP expression in the overall population (*P*_h_ = 0.327, I^2^ = 13.6%, crude HR = 1.652, 95% CI = 1.211–2.253) (Figure 3; Table 3), and even more so in the Asian population (*P*_h_ = 0.218, I^2^ = 30.6%, crude HR = 1.642, 95% CI = 1.195–2.257). The significant negative association between YAP expression and survival of patients with NSCLC was also observed in the overall population [disease-free survival (DFS): *P*_h_ = 0.878, I^2^ = 0.00%, crude HR = 2.562, 95% CI = 1.876–3.499; OS: *P*_h_ = 0.878, I^2^ = 0.00%, crude HR = 1.777, 95% CI = 1.233–2.562] and the Asian population (DFS: *P*_h_ = 0.414, I^2^ = 0.00%, crude HR = 2.515, 95% CI = 1.755–3.063; OS: *P*_h_ = 0.712, I^2^ = 0.00%, crude HR = 1.772, 95% CI = 1.214–2.587). Moreover, high *YAP*-mRNA was also significantly associated with the survival of patients with NSCLC (DFS: *P*_h_ = 0.920, I^2^ = 0.00%, crude HR = 2.617, 95% CI = 1.690–4.052). However, no association between them was observed in the multivariate combined analysis. High YAP expression was significantly associated with short OS of patients with SCLC in our combined multivariate analysis in the Asian population (*P*_h_ = 0.289, I^2^ = 11.10%, crude HR = 4.482, 95% CI = 2.182–9.209), but not with the crude data (*P*_h_ = 0.033, I^2^ = 78.00%, crude HR = 1.654, 95% CI = 0.434–6.300) (Figure 3; Table 3).

**Table 3:** The combined prognostic results of YAP in the meta-analysis.

**Biomarker**	**Disease**	**Survival**	**Crude HR and 95%CI**						**Adjusted HR and 95%CI**					
			**Race**	**Sample** **size**	**Eligible** **study**	***P***_h_**-value** **I^**2**^**	**Random** **model**	**Fixed** **model**	**Race**	**Sample** **size**	**Eligible** **study**	***P***_h_**-value** **I^**2**^**	**Random** **model**	**Fixed** **model**
YAP expression	Lung cancer	OS	Overall	642	6	0.327 13.6%	1.658 (1.172–2.345)	1.652 (1.211–2.253)	–	–	–	–	–	–
YAP	Lung cancer	OS	Asian	625	5	0.218 30.6%	1.655 (1.106–2.475)	1.642 (1.195–2.257)	Asian	442	5	0.000 89.3%	1.327 (0.480–3.667)	1.159 (0.847–1.587)
YAP expression	NSCLC	OS	Overall	345	4	0.878 0.0	1.777 (1.233–2.562)	1.777 (1.233–2.562)	–	–	–	–	–	–
YAP	NSCLC	OS	Asian	328	3	0.712 0.0%	1.772 (1.214–2.587)	1.772 (1.214–2.587)	Asian	343	3	0.00 89.8%	0.660 (0.211–2.061)	0.844 (0.595–1.196)
YAP expression	NSCLC	DFS	Overall	416	6	0.693 0.00	2.562 (1.876–3.499)	2.562 (1.876–3.499)	–	–	–	–	–	–
YAP expression	NSCLC	DFS	Asian	335	4	0.414 0.0%	2.515 (1.755–3.063)	2.515 (1.755–3.063)	Asian	343	3	0.001 85.3%	0.772 (0.332–1.795)	0.876 (0.648–1.184)
*YAP-*mRNA	NSCLC	DFS	Overall	136	3	0.920 0.0	2.617 (1.690–4.052)	2.617 (1.690–4.052)	–	–	–	–	–	–
YAP	SCLC	OS	Asian	297	2	0.033 78%	1.654 (0.434–6.300)	1.366 (0.759–2.459)	Asian	99	2	0.289 11.1%	4.465 (2.079–9.591)	4.482 (2.182–9.209)

*YAP means YAP protein expression detecting by immunohistochemistry and Western blotting; YAP expression includs YAP protein expression detecting by immunohistochemistry and Western blotting and YAP-mRNA transcription; NSCLC, non-small cell lung cancer; SCLC, small-cell lung cancer; Mixed race includes Asians and Caucasian cases; HR, hazards ratio; CI, confidence interval; P_h_-value, P-value of heterogeneity; OS, overall survival; DFS, disease–free survival*.

**Figure 4:**
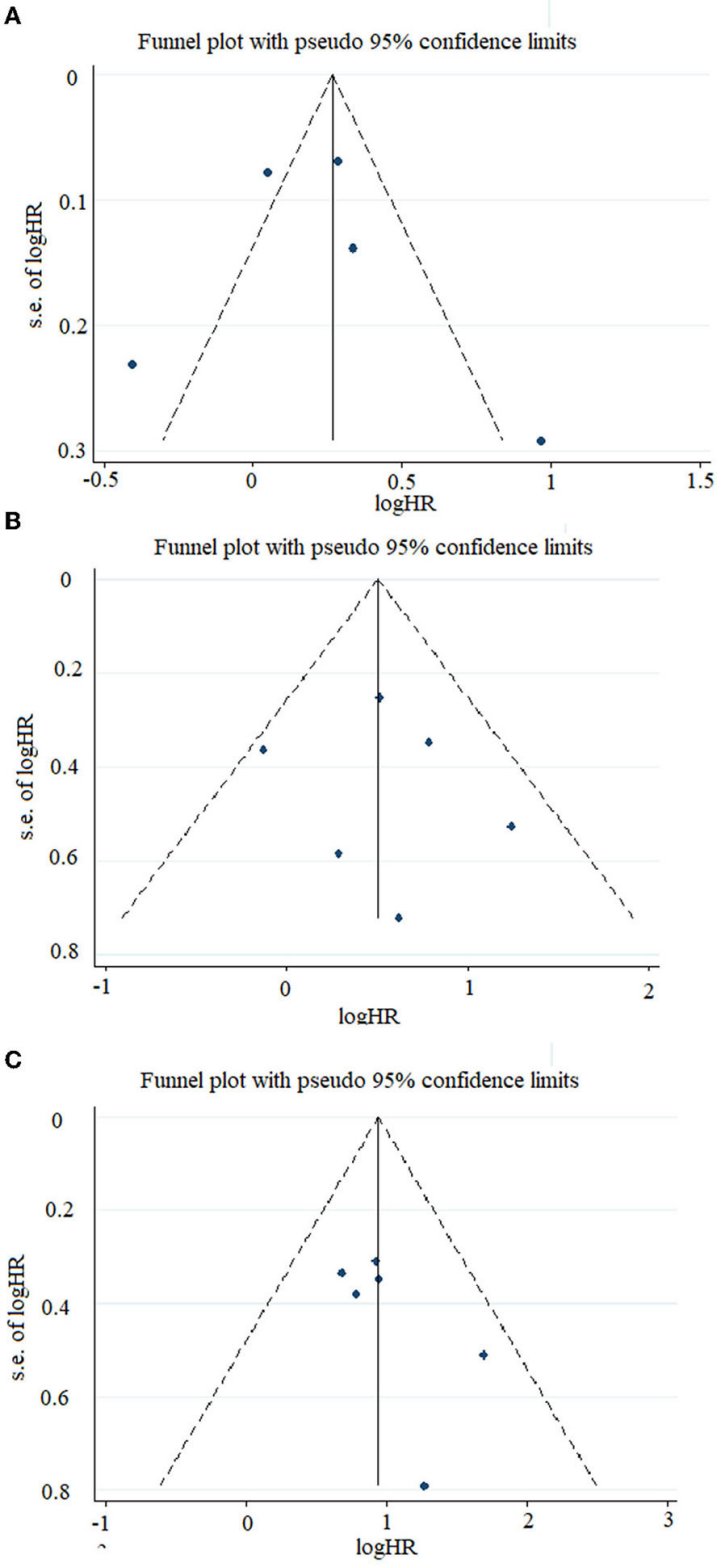
Funnel plots of the studies included in this meta-analysis. **(A)** Included studies related to TAZ expression and overall survival in the overall population. **(B)** Included studies related to YAP expression and disease-free survival in the overall population. **(C)** Included studies related to YAP expression and overall survival in the overall population.

In our study, relatively symmetric funnel plots were observed in our prognostic comparisons of patients with high- and low-TAZ NSCLC. Moreover, symmetric funnel plots were also found in the combined survival analysis of the patients with high- and low-YAP lung cancer, NSCLC, and SCLC (Figure 4).

## Discussion

The association of YAP and TAZ expression and lung cancer survival, particularly in patients with SCLC, remains unclear. This meta-analysis revealed that TAZ is significantly associated with poor OS of patients with NSCLC in the overall population and the Asian population. Moreover, a significant association was observed between high YAP expression and unsatisfactory survival of patients with lung cancer and patients with NSCLC, especially in the Asian population. However, no association between them was observed in the multivariate combined analysis. YAP expression was significantly associated with short OS in our combined analysis in the Asian SCLC population based on multivariate data, but not crude data.

Lung cancer is a complex and challenging disease that interacts with environmental and genetic factors (29). Many signaling pathways, including the Hippo pathway, are involved in lung cancer tumorigenesis and progression (30–34). YAP and TAZ are transcriptional co-factors. They are considered oncogenes in both NSCLC and SCLC (35, 36). YAP was found to mainly regulate lung cancer cells' growth and proliferation. In contrast, TAZ mainly promotes the migration of cancer cells (11). A recent meta-analysis performed by Feng et al. revealed that overexpression of TAZ is a predictive factor of poor prognosis and is associated with advanced TNM stage, poor tumor differentiation, and lymph node metastasis in various cancers (37). However, no association between TAZ expression and survival of patients with NSCLC was observed in a combined analysis of two studies (37). In our meta-analysis, TAZ was highly expressed in patients with NSCLC in most studies. The combined results showed that high TAZ expression is associated with a worse prognosis of patients with NSCLC in the overall and Asian populations. These findings suggest that TAZ is involved in NSCLC carcinogenesis and progression and that it is an independent prognostic factor of NSCLC.

Three meta-analyses reported the predictive role of YAP in clinical outcomes in various cancers (38–40). They found that both overall and nuclear YAP overexpression are intimately associated with worse OS and DFS in patients with malignancies (38, 40). Wu et al. reported no association between YAP expression and survival of lung cancer cases in their combined meta-analysis (40). However, a meta-analysis including six eligible studies showed that high nuclear expression of YAP1 was associated with shorter survival outcomes in patients with NSCLC (39). In the present study, we found an association between YAP expression and unsatisfactory survival of patients with lung cancer in the overall Asian population in our combined analysis with crude HR and 95% CI. Moreover, YAP expression at the protein and mRNA levels was significantly associated with the survival of patients with NSCLC using unadjusted HR and 95% CI. However, due to the small sample size and different confounding factors in each included study, we did not observe associations between them in combination with multivariate data. These findings demonstrate that YAP is an essential factor promoting the progression of lung cancer and NSCLC and that it is a novel prognostic factor for the disease. Due to the low number of studies in the Asian population in our combined analysis, we found no relationship between YAP expression and OS of patients with SCLC in our univariate analysis, whereas YAP expression was significantly associated with short OS of patients with SCLC in our combined analysis with multivariate data in the Asian population, indicating that YAP can also be considered a predictor of poor survival of patients with SCLC.

To the best of our knowledge, this meta-analysis used the largest sample size to date to analyze the prognostic roles of TAZ and YAP in lung cancer and patients with NSCLC. In addition, this is the first study to report the role of YAP in the prognosis of patients with SCLC. However, there are several limitations to this study. First, no studies on the association between TAZ expression and survival of patients with SCLC were included, so the prognostic role of TAZ in SCLC remains to be elucidated. Second, the sample size of included studies related to YAP expression in patients with SCLC was small. Our findings should be validated by multi-center clinical trials with larger sample sizes. It is also important to emphasize that most studies were conducted in the Asian population. Third, patients with different TNM stages and various treatment options were enrolled in each study. These factors might influence the combined results and hence, the results should be validated by prospective studies with patients with NSCLC or SCLC in specific TNM stages. Fourth, different expression models of YAP or TAZ, different detection methods, and different genetic backgrounds of the patients enrolled in the included studies might lead to inconsistent results.

In summary, *YAP* and *TAZ* function as oncogenes in both NSCLC and SCLC and aberrant protein expression levels of YAP and TAZ are independent and novel prognostic factors for these diseases. Further studies are warranted to validate the findings in the Asian population and explore effective biomarkers to predict the prognosis of patients.

## Author Contributions

YJ is responsible for the concept or design of the work. W-JX and R-WC are responsible for data collection. W-LY and HC are responsible for drafting the article. W-XC and J-PX are responsible for making important revisions to the article. All authors contributed to the article and approved the submitted version.

## Funding

The study was supported by the Fujian Provincial Natural Science Foundation (2018J01360).

## Conflict of Interest

The authors declare that the research was conducted in the absence of any commercial or financial relationships that could be construed as a potential conflict of interest.

## Publisher's Note

All claims expressed in this article are solely those of the authors and do not necessarily represent those of their affiliated organizations, or those of the publisher, the editors and the reviewers. Any product that may be evaluated in this article, or claim that may be made by its manufacturer, is not guaranteed or endorsed by the publisher.
